# Mucoepidermoid carcinoma of the middle ear: a case report

**DOI:** 10.3389/fsurg.2026.1779650

**Published:** 2026-03-31

**Authors:** Jianxin Hu, Yiyang Lu, Guochen Zhu

**Affiliations:** Department of Otorhinolaryngology-Head and Neck Surgery, Jiangnan University Medical Center (Wuxi No 2 People’s Hospital), Wuxi, China

**Keywords:** diagnosis, middle ear, mucoepidermoid carcinoma, pathology, treatment

## Abstract

Primary mucoepidermoid carcinoma of the middle ear is a rare malignancy. We report a case of 58-year-old woman who presented with purulent discharge from the left ear for seven months, followed by bloody otorrhea for one month. She was diagnosed with mucoepidermoid carcinoma of the middle ear after mastoidectomy. Then, she received x-ray radiation therapy (total dose: 3,000 cGy). However, nine months later, she underwent a comprehensive surgical intervention, including subtotal temporal bone resection, partial parotidectomy, and neck dissection, due to local tumor recurrence. The patient succumbed to tumor recurrence two years after the second surgery.

## 1 Introduction

Mucoepidermoid carcinoma (MEC), first characterized by Stewart and Becker in 1945, is histopathologically composed of varying proportions of mucous, epidermoid, and intermediate cells. Although MEC predominantly occurs in the major and minor salivary glands, it can occasionally occur in other glandular-lined structures, including the larynx, tracheobronchial tree, pharynx, esophagus, breast, and anal canal (1, 2). Primary MEC of the middle ear is an exceptionally rare entity, and reports in the literature are limited (3–7).

## 2 Case report

**Figure 1:**
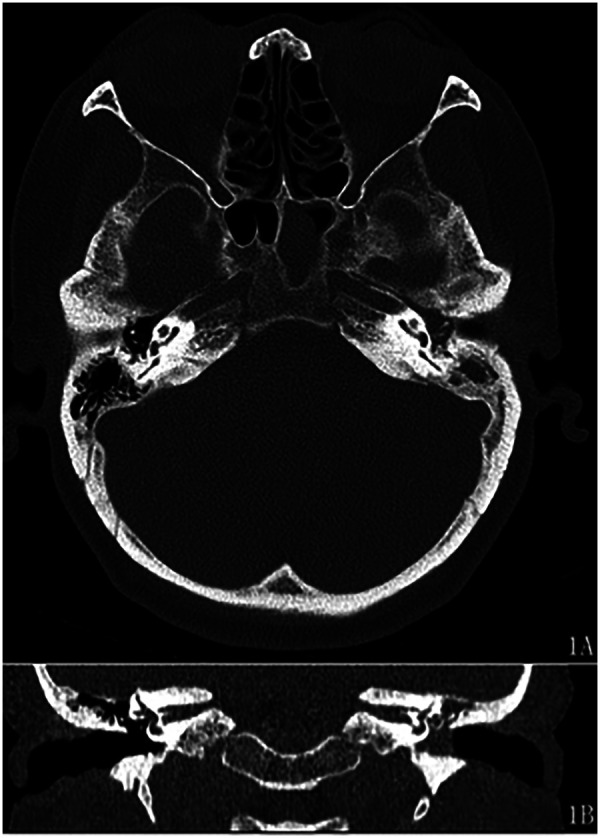
Temporal bone CT scans (axial and coronal views). Axial CT (**1A**) and coronal CT (**1B**): a soft tissue density mass occupying the left mastoid process, epitympanum, and mesotympanum.

A 58-year-old female with a seven-month history of recurrent purulent otorrhea in the left ear, which had progressed to bloody otorrhea one month prior to admission. The patient also reported associated ipsilateral hearing loss but denied tinnitus, vertigo, headache, vomiting or facial weakness. There was also no history of epistaxis or blood-stained postnasal drip. On physical examination, the left external auditory canal was patent. Otoscopy revealed a large perforation of the tympanic membrane with abundant edematous granulation tissue occupying the mesotympanum. There was no tenderness over the mastoid area. Facial nerve function was intact (House-Brackmann Grade I). Palpation of the parotid region revealed no masses, and no enlarged lymph nodes were detected in the neck. Electronic nasopharyngoscopy showed no abnormalities in the nasopharynx. Pure tone audiometry demonstrated moderate conductive hearing loss in the left ear. Temporal bone computed tomography (CT) scans (axial and coronal views) revealed a soft tissue density mass occupying the left mastoid process, epitympanum, and mesotympanum. And the imaging also demonstrated clear dural planes and intact bony cortices of the skull base without evidence of erosion, infiltration, or abnormal enhancement. (Figure 1). Preoperative biopsy of the middle ear lesion revealed high-grade squamous intraepithelial neoplasia. The patient was admitted with a working diagnosis of left middle ear tumor.

**Figure 2:**
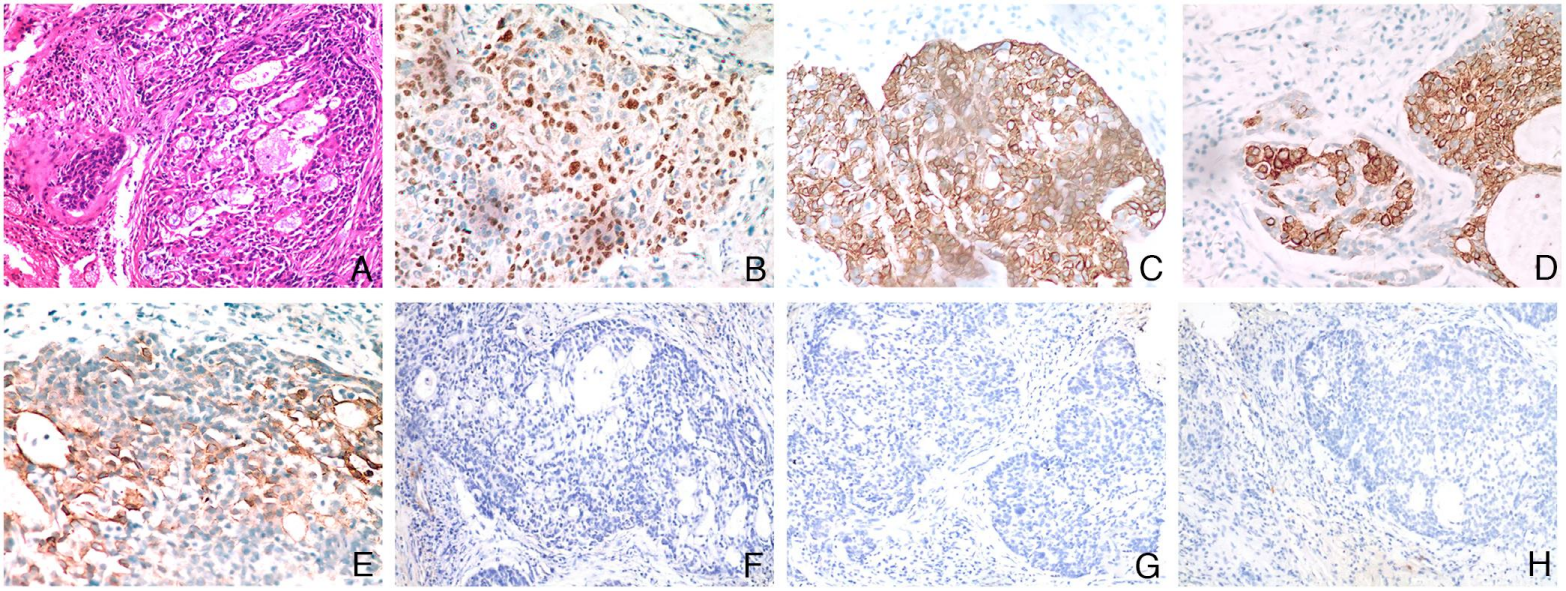
Histopathological examination. **(A)** The tumor is composed of an admixture of mucous, epidermoid, and intermediate cells, typically arranged in nests or forming glandular lumens (H&E, ×100 magnification); **(B–E)** Tumor cells exhibit positive immunoreactivity for P63, HMWC, CK8, and CK18 (EnVision method immunohistochemical staining, ×200 magnification); **(F–H)** Tumor cells demonstrate negative immunoreactivity for Actin, Calponin, and CD117 (EnVision method immunohistochemical staining, ×100 magnification).

Under general anesthesia, a left mastoidectomy was performed. During the operation, numerous and extensive growths exhibiting a granulation tissue-like appearance were observed occupying the mastoid, epitympanum, and mesotympanum. The Eustachian tube was patent, but partial destruction of the tegmen tympani was noted. Importantly, there was no involvement of the facial nerve, semicircular canals, or vestibule. The patient has recovered well after the operation. Histopathological examination of the surgical specimen, performed with hematoxylin-eosin (H&E) staining, confirmed the presence of MEC, characterized by a admixture of mucous, epidermoid, and intermediate cells. (Figure 2A). Immunohistochemical analysis was performed using the EnVision method. The results confirmed the biphasic differentiation typical of mucoepidermoid carcinoma: epidermoid and intermediate cells demonstrated nuclear and cytoplasmic positivity for P63 and high molecular weight cytokeratin (HMWCK), respectively. (Figures 2B,C). CK8 and CK18 positivity highlighted mucous cells with clear cytoplasm against a diffuse background. (Figures 2D,E). Importantly, the tumor cells were uniformly negative for Actin, Calponin, and CD117, which further supported the diagnosis and helped exclude other entities, such as pleomorphic adenoma, adenoid cystic carcinoma and other tumors that originate from salivary glands but may occasionally be found in the middle ear. (Figures 2F–H). The definitive pathological diagnosis was mucoepidermoid carcinoma of the middle ear. The immunohistochemical analysis indicated the tumor with a prominent cystic component and an absence of angiolymphatic or perineural invasion, mitotic activity was low, supporting its classification as low-grade. However, the patient declined further extensive surgical resection and subsequently received radiotherapy at a total dose of 3,000 cGy.

Nine months following the initial surgery, the patient presented with local recurrence of the middle ear tumor. The patient exhibited no facial nerve palsy and no evidence of regional lymph node. Distant metastasis was excluded based on negative findings on chest CT, abdominal and pelvic ultrasound, and whole-body bone scintigraphy. Under general anesthesia, a comprehensive salvage surgery was performed, consisting of subtotal temporal bone resection, partial parotidectomy, and cervical lymph node dissection (levels I-III). Intraoperatively, the previous mastoidectomy cavity was found to be completely filled with recurrent neoplastic growths. The tumor was observed to be adherent to the dura mater in the tegmen area, in close proximity to the geniculate ganglion of facial nerve, and had invaded the bony segment of the Eustachian tube. Furthermore, approximately 0.5 cm of the internal carotid artery was partially exposed, situated anterior and inferior to the cochlea. Postoperative pathological findings were consistent with those of the initial surgery, confirming a low-grade MEC. Significantly, the excised parotid gland and cervical lymph nodes were free of tumor involvement, and all resection margins were microscopically clear. Despite these favorable surgical findings, the patient declined further adjuvant therapy and unfortunately succumbed to recurrent disease two years following the second surgical intervention.

## 3 Discussion

Malignant tumors of the middle ear, as a rare and insidious malignant tumor in the base of the skull, have early clinical manifestations highly similar to those of chronic otitis media, leading to delayed diagnosis and subsequently affecting the prognosis of patients. In contrast to non-specific symptoms such as otalgia, otorrhea, and hearing loss, which are frequently observed and often confused with benign middle ear pathologies, the presence of more indicative clinical manifestations like bloody otorrhea, facial nerve involvement, and cervical lymphadenopathy serves as critical warning signs for middle ear malignancies. These specific findings necessitate heightened clinical vigilance and prompt diagnostic evaluation. Squamous cell carcinoma is the predominant type of middle ear malignancy histopathologically, but there are also sporadic reports of basal cell carcinoma and adenoid cystic carcinoma. Squamous cell carcinoma is characterized microscopically by uniform epidermoid cells forming solid nests, with keratin pearl formation and lack of mucous cells, it is typically negative for CK8/18 immunohistochemically (8). Adenoid cystic carcinoma, although capable of expressing CK8/18 and P63, is distinguished by a basaloid P63 distribution around cribriform structures, pseudocysts containing myxoid stroma rather than true mucous cells, and prominent perineural invasion that often manifests clinically as early-stage intermittent otalgia. Basal cell carcinoma is rare and typically indolent, composed of basaloid cells arranged in palisading patterns with basement membrane material deposition highlighted by PAS staining, and lacks mucous cell components (9). Notably, primary MEC of the middle ear is an exceedingly rare entity, and accurate pathological grading plays a crucial role in evaluating and predicting the long-term prognosis of patients.

The precise pathogenesis of middle ear MEC remains elusive, Soh et al. (3) have proposed several hypotheses regarding its origin. Firstly, the middle ear mucosal epithelium may undergo metaplastic changes, accompanied by an aberrant proliferation of glandular components, possibly as a result of chronic middle ear disease stimulation. Secondly, the epidermoid component of the tumor may arise from squamous metaplasia induced by chronic middle ear inflammation. Thirdly, during embryonic development, seromucinous or minor salivary gland tissue may ectopically persist in the middle ear region; indeed, studies have indicated the presence of seromucinous glands with low secretory activity in the cartilaginous portion of the Eustachian tube. Fourthly, the tumor could also result from direct invasion of the middle ear by MEC originating from adjacent organs or tissues (e.g., lesions of the parotid gland, external auditory canal, or parapharyngeal minor salivary glands). In the present case, our findings support a primary origin within the middle ear and mastoid. Given that the examinations of the external auditory canal, nasopharynx, and parotid area were all normal, with no mass detected in the parapharyngeal infratemporal fossa, the possibility of a secondary MEC can therefore be definitively excluded.

MEC is characterized by a variable admixture of three distinct cell types: mucous cells with clear to vacuolated cytoplasm, epidermoid cells with eosinophilic cytoplasm, and intermediate cells with scant cytoplasm. These cells are arranged in solid nests, cystic spaces, or duct-like structures, the ratio of cystic to solid components and the degree of cytologic atypia are both associated with tumor grade. MEC is predominantly classified into three malignancy grades: low, intermediate, and high. Various grading systems, including the AFIP grading system, the Brandwein system, the modified Healey system, and the MSK system, provide frameworks for evaluating tumor characteristics based on crucial histopathological parameters. These parameters encompass the ratio of cystic to solid components, presence of nerve invasion, extent of cell necrosis, cellular mitotic activity, and intercellular alterations (1, 10). Low-grade MEC typically presents with well-defined gross boundaries, exhibiting rare instances of local invasion and distant metastasis. Histologically, these tumors are marked by a predominance of well-differentiated mucous cells, often forming cystic cavities of varying sizes. In contrast, highly malignant cases mainly consist of epidermoid cells and intermediate cells, with fewer mucus cells. Intercellular degeneration is obvious, and mitosis can be observed. Tumors often form solid epithelial masses and are prone to infiltrate peripheral nerves, blood vessels or lymph. Those with moderate malignancy fall between the above two categories. Significantly, literature reveals that while the majority of middle ear MEC cases are reported as highly malignant (5–7, 10), most instances of salivary gland MEC are classified as low malignancy. This inconsistency merits further investigation into the biological behavior of MEC in different anatomical locations, as it may have significant implications for clinical management and prognosis. Understanding these variances could enhance our approach to patient treatment strategies and highlight the need for tailored diagnostic criteria in different tissues.

More than half of the MECs, especially in the low and moderate grades, have characteristic gene fusions, which are key targets for diagnosis and research. The translocation t(11; 19)(q21; p13), which results in the CRTC1-MAML2 fusion oncogene (11), is currently recognized as a highly specific diagnostic biomarker for MEC, and this gene is often closely related to the prognosis of cancer (12). The detection of MAML2 gene rearrangement is particularly valuable in distinguishing MEC from other salivary gland neoplasms with overlapping histological features (11). In cases where morphological and immunohistochemical findings are equivocal, the detection of MAML2 gene rearrangement serves as an invaluable ancillary tool. While our case presented with classic histological features, future diagnosis and prognosis for middle ear tumors would benefit from the integration of molecular analysis.

The primary treatment MEC is radical surgical resection, often supplemented by adjuvant radiotherapy. Patient prognosis is primarily dictated by pathological grade, clinical stage, and the adequacy of surgical resection (2–7, 10). Given the rarity, aggressive behavior, and anatomical complexity of middle ear MEC, a multidisciplinary team (MDT) approach, involving otologists, head and neck surgeons, oncologists, radiation oncologists, and pathologists, is crucial to optimize individualized treatment planning and patient management. Consistent with treatment strategies for other malignant neoplasms of the middle ear, the surgical management of middle ear MEC may involve mastoidectomy, extended subtotal temporal bone resection, or extended total temporal bone resection, with selective cervical lymph node dissection performed as indicated (13, 14). Adjuvant chemoradiotherapy is frequently administered for advanced cases or those with high-risk features. Furthermore, rigorous and systematic long-term follow-up, incorporating regular clinical examinations and imaging studies, are imperative for the early detection of local or regional recurrence, distant metastasis, and treatment-related complications, especially considering the high recurrence potential of MEC. In the present case, the initial treatment consisted of a mastoidectomy followed by postoperative radiotherapy. Subsequently, the patient underwent a second surgical intervention comprising subtotal temporal bone resection, partial parotidectomy, and cervical lymph node dissection.

## 4 Conclusion

The following experiences during the diagnosis and treatment of this case are worth learning from: (1) Although primary malignant tumors of the middle ear are relatively rare, cases with bloody otorrhea should be highly vigilant (7). (2) Middle ear biopsy has limitations such as small sample size and superficial sampling, which may lead to misdiagnosis or missed diagnosis. If a middle ear malignant tumor is highly suspected, rapid section examination should be performed as much as possible during the operation. Even if the preoperative biopsy report is negative, the possibility of mucoepidermoid carcinoma should be considered in addition to common squamous cell carcinoma and adenoid cystic carcinoma. (3) The first treatment plan has a significant impact on prognosis. If subtotal temporal bone resection or/and radical radiotherapy (dose 6,000–7,000 cGy) was performed shortly after mastoidectomy in this case, a better treatment effect might have been achieved. (4) Due to factors such as the tumor being wrapped by the temporal bone and complex adjacent structures, complete surgical resection is difficult. Therefore, postoperative radiotherapy can improve the therapeutic effect; if the lesion involves important structures such as the carotid canal, radiotherapy can be considered first, and then surgery can be performed after the tumor range is reduced (13, 14). (5) The risk of recurrence is high after treatment, lifelong and regular follow-up is required.

## Data Availability

The original contributions presented in the study are included in the article/Supplementary Material, further inquiries can be directed to the corresponding author.
